# Human Lsg1 defines a family of essential GTPases that correlates with the evolution of compartmentalization

**DOI:** 10.1186/1741-7007-3-21

**Published:** 2005-10-07

**Authors:** Emmanuel G Reynaud, Miguel A Andrade, Fabien Bonneau, Thi Bach Nga Ly, Michael Knop, Klaus Scheffzek, Rainer Pepperkok

**Affiliations:** 1 Cell Biology and Cell Biophysics Programme, European Molecular Biology Laboratory (EMBL), Meyerhofstrasse 1, 69117 Heidelberg, Germany; 2 Ontario Genomics Innovation Centre, Ottawa Health Research Institute, 501 Smyth, Ottawa, ON K1H 8L6, Canada; 3 Structural and Computational Programme, European Molecular Biology Laboratory (EMBL), Meyerhofstrasse 1, 69117 Heidelberg, Germany

## Abstract

**Background:**

Compartmentalization is a key feature of eukaryotic cells, but its evolution remains poorly understood. GTPases are the oldest enzymes that use nucleotides as substrates and they participate in a wide range of cellular processes. Therefore, they are ideal tools for comparative genomic studies aimed at understanding how aspects of biological complexity such as cellular compartmentalization evolved.

**Results:**

We describe the identification and characterization of a unique family of circularly permuted GTPases represented by the human orthologue of yeast Lsg1p. We placed the members of this family in the phylogenetic context of the **Y**lqF **R**elated **G**TPase (YRG) family, which are present in Eukarya, Bacteria and Archea and include the stem cell regulator Nucleostemin. To extend the computational analysis, we showed that hLsg1 is an essential GTPase predominantly located in the endoplasmic reticulum and, in some cells, in Cajal bodies in the nucleus. Comparison of localization and siRNA datasets suggests that all members of the family are essential GTPases that have increased in number as the compartmentalization of the eukaryotic cell and the ribosome biogenesis pathway have evolved.

**Conclusion:**

We propose a scenario, consistent with our data, for the evolution of this family: cytoplasmic components were first acquired, followed by nuclear components, and finally the mitochondrial and chloroplast elements were derived from different bacterial species, in parallel with the formation of the nucleolus and the specialization of nuclear components.

## Background

Comparative genomics is a powerful method for identifying the potential functions of previously uncharacterized genes, allowing their distribution among the kingdoms of life to be characterized, and the changes in sequence and regulation underpinning their conserved or divergent functions to be tracked [1]. Comparative genomics has been enormously facilitated by progress in bioinformatics tools, comprising the enormous amount of information available from databases concerning protein localization [2,3], viability [4,5], protein expression [6], genetic interactions [7] and protein-protein interactions [8]. These resources are usually focused on one particular organism (*S. cerevisiae*, *C. elegans*, *D. melanogaster *or *B. subtilis*) and are therefore mainly used by the small part of the scientific community working with this organism and able to handle the outcome and limitations. Attempts have been made to correlate large datasets across species, for example in the case of protein-protein interactions [9]. These cross-correlation analyses are based on the presumption that sequence and structural similarities between gene products can be used to assess functional similarities [10,11] and could in principle be extended to protein localization, viability or partners.

Genomics should be particularly powerful in the case of GTP binding proteins (or GTPases), which despite extraordinary functional diversity are all believed to have evolved from a single common ancestor [12]. As a result, all known GTPases have a conserved switch mechanism of action, core structure and sequence motifs. These proteins are found in all domains of life and are involved in such essential processes as vesicular trafficking, protein translation, intracellular signal transduction and cell cycle progression [12-14]. GTP binding proteins are often described as molecular switch proteins because of their particular mode of action. Binding and hydrolysis of GTP results in conformational changes in the so-called switch regions of the protein, which define the active GTP- and the inactive GDP-bound forms; these are used, for instance, for regulating receptor activation and cargo recruitment to membranes [12].

We have used comparative genomics to identify and characterize the human homologue of the yeast protein Lsg1. Here, we describe a novel family of GTP binding proteins, which we have named YRG (YlqF Related GTPases). Members of this family contain a central GTPase domain showing a unique circular permutation of the known G motifs of the GTP binding proteins. A phylogenetic analysis was used for cross-species comparisons, focusing on sub-cellular localization, cell viability and the known functions of each subfamily member. This analysis showed that YRG family members are essential, have increased in eukaryotes as cell compartmentalizationhas evolved, and show functional conservation in relation to rRNA maturation.

## Results

Recently, we have localized more than 800 human proteins in living cells with the aim of gaining preliminary functional data [3]. Analysis of these proteins for sequences exhibiting characteristic GTPase motifs such as the P-loop [18] allowed us to identify a subset of proteins as putative GTPases.

### Human Lsg1p defines a highly conserved GTPase protein family

**Figure 1:**
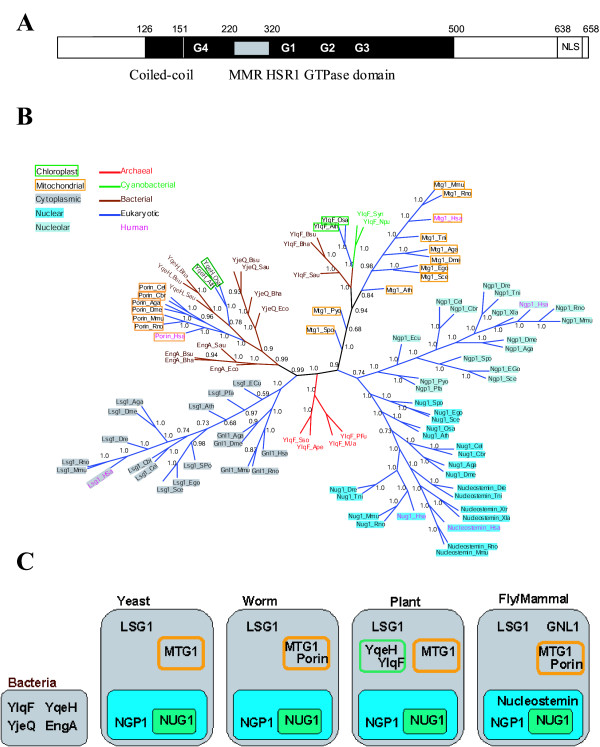
**hLsg1 is member of a large circularly permuted GTPase family. **(A) **Schematic representation of hLsg1 **– hLsg1 encodes a protein of 658 amino acids comprising a central MMR/HSR1 GTPase domain (black box, 151–500), a coiled-coil domain (Hatched box, 126–151) and a nuclear localization signal (NLS, grey box, 638–654). Domain organization of the GTPase is indicated as well as the insertion (white box inside the black box, 220–320) separating the G4 motif from the G1 motif. (B) **Phylogenetic tree of the YRG superfamily. **We constructed a multiple alignment of representative sequences from the YqeH, YjeQ, EngA, and YlqF families. The alignment was produced using ClustalW followed by manual editing [16]. The tree was generated from the alignment using MrBayes v3 [45] (100000 generations with parameter n chains = 4; convergence occurred after 33600 generations; the tree is the consensus of 664 trees computed using MrBayes. No molecular clock was assumed and therefore the branch lengths have no meaning. The numbers indicate the fraction of trees displaying the grouping given by the branch). The root of the tree is the one given by the MrBayes output. (C) **Distribution of YRG members in cellular compartments in different organisms.**

One of these proteins possesses a central GTPase domain defined in the PFAM database of protein domains [19] as the MMR/HSR1 domain, a coiled coil, and a potential nuclear localization signal (NLS) (Figure 1A). No additional structural or enzymatic domains could be identified in the protein sequence using the SMART domain research server [20]. Interestingly, this GTPase domain is circularly permuted [21], in contrast to the canonical organization of GTPases based on the small GTPase Ras [12,22]. This circular permutation is unique, and it is surprising in view of the structure of the GTPase P-loop domain [22,23]. It implies that the four highly conserved elements of the GTPase that mediate interactions with the guanine nucleotides and effector proteins, known as the G1, G2, G3 and G4 motifs, are circularly permuted and reorganized, since G4 is followed by G1, G2 and G3 [12,22] (Figure 1A).

A BLAST search for similar protein sequences [15] shows that this unusual GTPase is present as a single copy per genome [22] (Figure 1B [see Additional file 1]). Only one member of the family has so far been experimentally defined, namely the Lsg1 protein in *S. cerevisiae *[24]. Accordingly, we named the human protein hLsg1 (**h**uman orthologue of **Lsg1**). All orthologues of hLsg1 possess a central MMR/HSR1 domain belonging to KOG1424 in the database of clusters of orthologous genes [25]. The identity between aligned sequences ranges from 31% to 88%. Interestingly, except in the *E. cuniculi *member, the GTPase domain contains an unusual insertion in comparison to the canonical GTPase structure. This insertion separates the G4 element from the remaining GTPase elements (G1, G2 and G3) (Figure 1A).

In order to elucidate the potential function of hLsg1, we extended our phylogenetic analysis. Owing to their unique structure, circularly permuted GTPases have previously been reported [22,23] and partially grouped into the Yawg/YlqF family (COG1160) [26], which is mainly restricted to prokaryotes and microbial eukaryotes (*S. cerevisiae*, *E. cuniculi*). This family contains five subfamilies: YjeQ (YloQ), MJ1464, YqeH, YlqF and Yawg. The latter three branches have eukaryotic members, YlqF representing the ancestor of hLsg1. Interestingly, while the YqeH subfamily is limited to only one member per species (also labeled as Euk-porin in sequence database), and the YjeQ subfamily is mainly restricted to bacteria [22], the YlqF subfamily shows a large expansion of this gene family in eukarya (Figure 1C). The YlqF subfamily can be further subdivided into five clades: YlqF (bacterial), MTG1 (KOG2485), LSG1 (KOG1424), NOG2 (Yawg, KOG2423), and NUG1 (KOG2484) according to the *S. cerevisiae *nomenclature. The YlqF family expands further in Coelomates [GNL1, 23] and in Deuterostomia (Nucleostemin [27]) (Figure 1B [see Additional file 2]).

Next, we exploited the experimental data from a comprehensive large-scale localization screen in yeast [2] and we conducted literature searches to deduce the possible cellular localizations of the different family members, ranging from the nucleolus to the mitochondria. The nucleolus is the compartment in which the large ribosomal RNA precursor (pre-rRNA) is synthesized, processed into the mature 18S, 5.8S, and 28S rRNAs and assembled with proteins to form ribosomal subunits that move to the nucleoplasm and are finally exported to the cytoplasm. Mitochondria and chloroplasts also possess a set of ribosomes. All yeast members (LSG1, NOG2, NUG1 and MTG1) are involved in ribosome biogenesis [24,28-30], and YjeQ binds to the ribosome in *E. coli *[31]. Finally, using ChloroP [32] to predict proteins localized to the chloroplast, we detected a sixth subfamily in YlqF, called ChYlqF (for Chloroplast YlqF), and a second subfamily in YqeH, called ChYqeH (for chloroplast YqeH). These are only found in plant genomes and group in the phylogenetic tree with the cyanobacteria YRG and YqeH members (Figure 1B [see Additional files 1 and 2]).

### Nucleotide binding and GTPase activity of hLsg1

**Figure 2:**
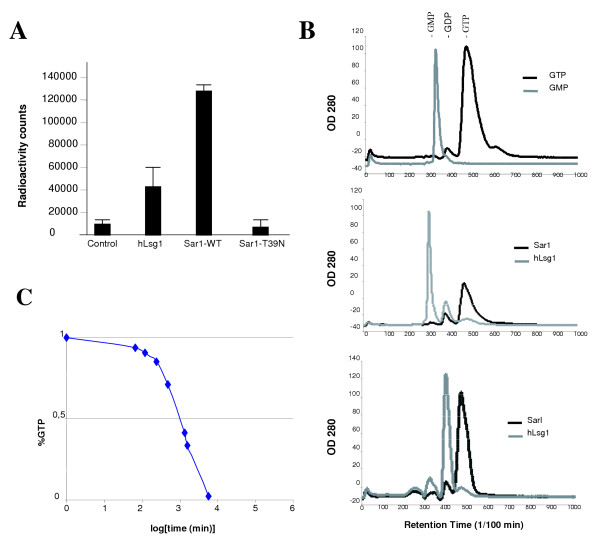
**Nucleotide binding and GTPase activity of hLsg1. **(A) **GTP binding of hLsg1. **Nucleotide binding was measured as described in Materials and Methods. BSA was used as control, while Sar1p-WT and the GDP-restricted Sar1p mutant (Sar1p-T39N) were used as positive and negative GTP binding controls, respectively. The graph is the sum of three separate experiments. (B) **GTPase activity of purified recombinant and immunoprecipitated hLsg1**. Elution times of GDP and GTP standards are indicated (*top panel*). GTPase activities of purified recombinant Sar1-WT and hLsg1 are shown (*middle panel*) as well as GTPase activities of immunoprecipitated Sar1p and hLsg1 (*lower panel*). Incubation times were identical (18 h) except for the hLsg1 precipitate (4 h). (C) **Hydrolysis of GTP by hLsg1**. GTPase activities of purified recombinant hLsg1 were analyzed by HPLC as described in Materials and Methods. A solution containing 5 μM hLsg1 and 200 μM GTP was incubated at 37°C. Samples were taken at different time-points and analyzed for percentages of GTP and GDP.

Lsg1-related proteins contain motifs that have been found to be important for guanine nucleotide binding and GTPase activity in a variety of cellular proteins [33]. Lsg1-related proteins contain the G1-4 motifs typical of GTPases (Figure 1A), suggesting that members of this family are likely to exhibit guanine nucleotide binding and GTPase activity. However, direct experimental evidence for this has been lacking so far, except for the distantly related bacterial homologues YjeQ [31], YlqF and YqeH [34]. To test this function in human Lsg1, we examined the binding of [^32^P] GTP to purified His tagged-hLsg1 using as control a His-tagged Sar1p, a well characterized GTPase regulating the vesicular coat complex COPII [35,36]. As shown in Figure 2A, hLsg1 binds to [^32^P] GTP, although more weakly than Sar1p. However, hLsg1 did not bind GDP under those experimental conditions (data not shown), which may reflect weak binding. To determine the GTPase activity of hLsg1, we performed a GTPase assay using an HPLC system as previously described [17]. In this assay, purified recombinant hLsg1 showed a low GTPase activity that proceeded to GMP and induced the further hydrolysis of GDP through GMP to guanosine. Such low GTPase activities have previously been observed in the distantly related bacterial homolog YjeQ [21], but also in GTPases in general, since their activities rely heavily on co-factors such as GAPs (GTPase Activating Proteins) [41] or GEFs (Guanine nucleotide Exchange Factors) [37]. Moreover, other GTPases such as the interferon-induced 67-kDa guanylate-binding protein (hGBP1) have been shown not to limit their hydrolysis to GDP [42]. To confirm our observations indicating that hLsg1 has GTPase activity, we immunoprecipitated endogenous hLsg1 from a HeLa cell extract using a polyclonal antibody raised against purified hLsg1, and analyzed the GTPase activity of the precipitate (Figure 2B). The GTPase activity was four times higher (incubation time 4 h compared to 18 h required for completion of GTP hydrolysis) than that of *in vitro *purified recombinant hLsg1 and GDP was the only final product (Figure 2B, lower panel). These data demonstrate GTPase activity in a eukaryotic member of the YlqF family for the first time.

### hLsg1 is an essential protein, a characteristic of YRG family members

**Figure 3:**
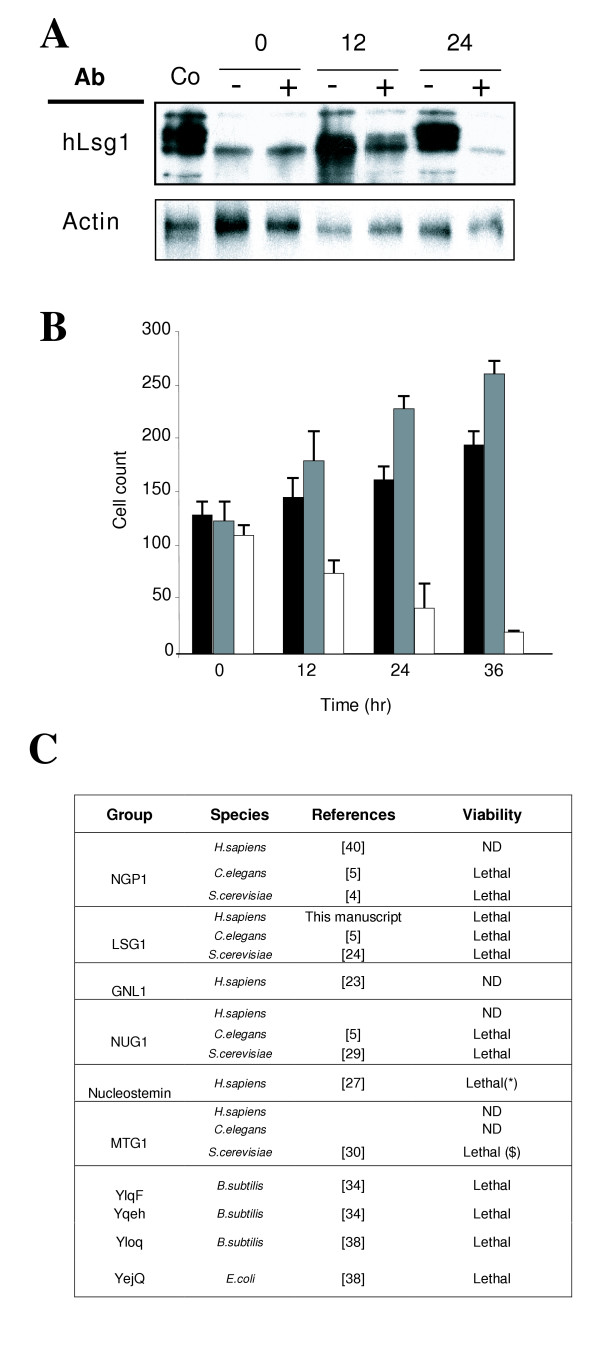
**hLsg1 is an essential protein. **(A) **Rapid disappearance of hLsg1 in cells transfected with hLsg1 specific siRNA**. HeLa cells were transfected with an hLsg1-specific siRNA (+) or with a scrambled siRNA as a negative control (-), and were harvested at 0, 12 and 24 h post-transfection. Extracts were prepared and 30 μg of each sample were separated on an 8% polyacrylamide gel and analyzed for hLsg1 by western blotting. Untreated cell lysate (30 μg) from confluent HeLa cells was run as a control (Co). To assess the specificity of the siRNAs, 30 μg of each extract was run on a 12% SDS-PAGE gel and analyzed for actin content by western blotting. (B) **Cell count. **HeLa cells plated on coverslips and transfected with no siRNA (black box), hLsg1-specific siRNA (white box), or a scrambled siRNA (grey box) were fixed at 0, 12, 24 and 36 h post-transfection. Cells were labeled with Dapi and anti-hLsg1 antibodies and the cell number was counted. The graph is the sum of three independent experiments (C) **YRG family member lethality. **Literature survey and database searches indicating that YRG family members are essential.

Yeast Lsg1, like the yeast YRG homologues NUG1, NOG2 and MTG1, is an essential protein [4]. To confirm the consequences of loss of hLsg1, we transfected siRNAs targeted against hLsg1 into HeLa cells, confirming the efficiency of the siRNA treatment by western blot analysis (Figure 3A). After 24 h, Lsg1 expression showed a drastic decrease in cells treated with Lsg1 siRNA compared to cells treated with a negative control siRNA. Moreover, hLsg1 expression in control cells or cells transfected with the negative control shows a band shift that increases with time, as observed in proteins post-translationally modified e.g. by phosphorylation. The increase in intensity could indicate that the polyclonal antibody has a higher affinity for the modified form. There was no significant change in actin expression in control cells, or in cells treated with either random siRNA or a specific hLsg1 siRNA (Figure 3A). However, microscopic observations during the course of the experiment showed that HeLa cell cultures exhibiting hLsg1 knockdown were less dense than control cells. In addition, hLsg1 knockdowns contained more apoptotic cells (not shown), suggesting a lethal effect. We confirmed this by immunostaining hLsg1-specific siRNA-treated cells with a polyclonal anti-hLsg1 antibody and staining the cell nuclei with DAPI at different times after transfection of the siRNA (Figure 3B). Cell numbers decreased rapidly after treatment with the specific hLsg1 siRNA in comparison to cells treated with oligofectamine alone or with control siRNA.

We used the large datasets from gene viability screens of bacteria, worms and flies to compare our observations with data about other YRG family members. YjeQ was shown to be indispensable for the growth of *E. coli *and *B. subtilis *[38]. In *C. elegans, YRG *orthologues are non-viable (t19a6.2a, t19a6.2b, k01c8.9, C53H9) (Figure 3C). Since large human RNAi screens are only now in progress, no data were available for other YRG human genes. However, interestingly, overexpression of nucleostemin was shown to be lethal [27].

According to our results, hLsg1 is essential, like its yeast counterpart, and this characteristic seems to be common to the YRG family members. This implies that each YRG protein fulfils essential functions.

### hLsg1 localizes to the endoplasmic reticulum and to discrete nuclear structures

Compartmentalization of the human cell allows better control of function and reactions steps in many pathways, including ribosome assembly. Cellular localization is a key to defining protein function. Using large-scale localization screens, we previously identified hLsg1 as an endoplasmic reticulum localized protein [3], in contrast to yeast Lsg1, which is proposed to localize specifically to the cytosol [24].

**Figure 4:**
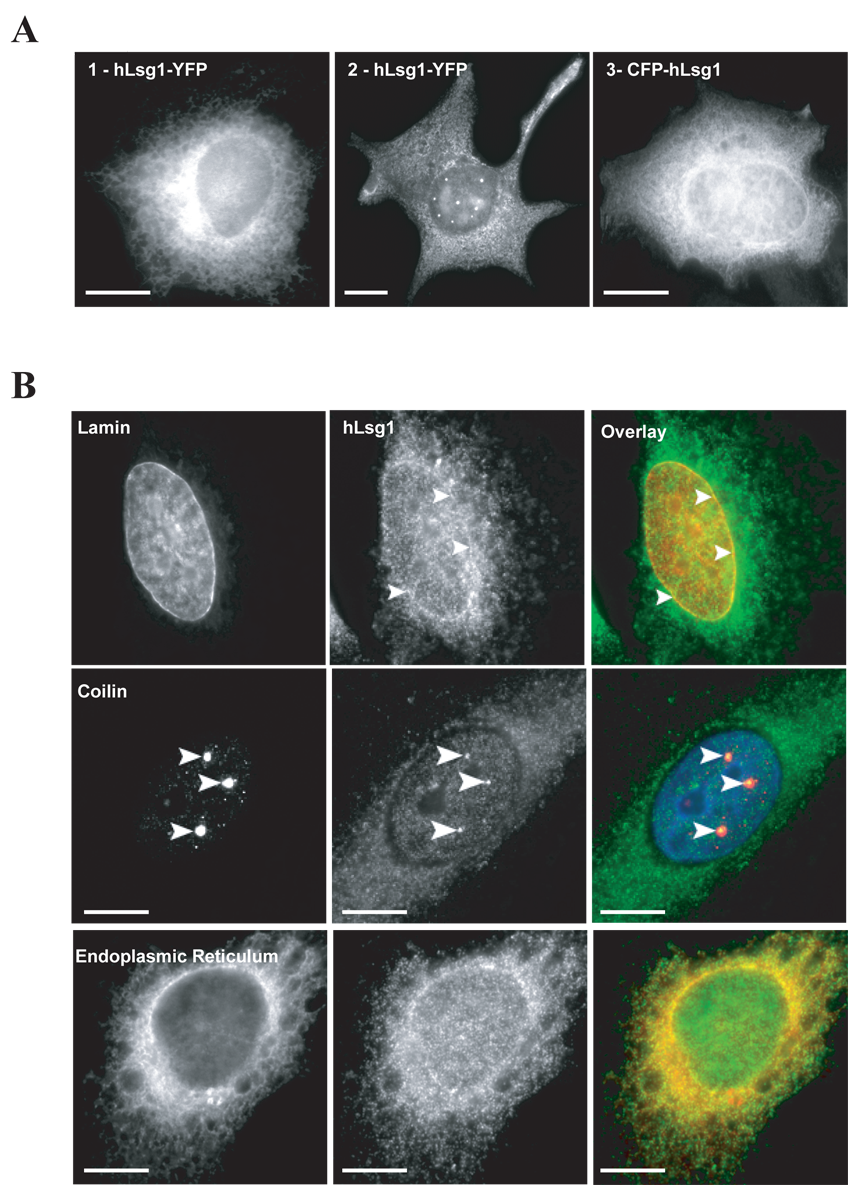
**Subcellular localization analysis of hLsg1. **(A) **Subcellular localization of YFP and CFP tagged hLsg1 **– HeLa cells were transiently transfected with hLsg1-YFP (1, 2), or CFP-hLsg1 (3) and visualized by fluorescence microscopy. (B) **Localization of endogenous hLsg1**. hLsg1 was visualized by staining with anti-hLsg1 antibodies, followed by Alexa 488-conjugated anti-rabbit antibodies. HeLa cells were transiently transfected with YFP-tagged lamin or Clontech ER-YFP marker. Coilin was visualized by monoclonal mouse anti-coilin antibodies, followed by rhodamine-conjugated anti-mouse antibodies. Bars = 10 μm

We decided to confirm our preliminary data on hLsg1 localization in humans using GFP-fused constructs as well as specific polyclonal antibodies. When expressed as a C-terminally tagged YFP fusion protein, hLsg1 localized to the ER in most cells (Figure 4A, 1). In 10% of the transfected cells, however, discrete structures in the nucleus were observed and localization to the endoplasmic reticulum was decreased or even absent (Figure 4A, 2). An N-terminally tagged CFP-hLsg1 fusion protein was also localized to the ER and nuclear envelope, but more of the protein was cytosolic than in the case of the C-terminal hLsg1-YFP fusion (Figure 4A, 3). A truncated hLsg1 version (480 to 658aa) fused to the YFP, containing the potential NLS, accumulated in the nucleus and nucleolus (Figure 4A, 4). Collectively, these data indicate that the NLS present in the C-terminus of hLsg1 is functional, in contrast to the putative NLS in yeast Lsg1, which is reportedly restricted to the cytosol [24].

Immunostaining with an antibody against the entire protein showed that the endogenous protein also localized to reticular membranes, and in a fraction of the cells to a number of small punctuate nuclear structures. These results are very similar to those obtained with the hLsg1-YFP fusion protein (Figure 4A, 2). Double staining showed that hLsg1 partially co-localized with an ectopically expressed FP (fluorescent protein) used to mark the ER *(Clontech ER marker) *(Figure 4B, bottom row), as well as with the nuclear envelope marker lamin B1 (Figure 4B, top row). However, hLsg1 was largely absent from the Golgi complex, which was labeled with antibodies against the Golgi membrane protein golgin97, and from mitochondria, marked by antibodies against HSP60 (data not shown). Moreover, the small hLsg1-positive nuclear structures observed in a fraction of the cells co-localized with coilin, a typical marker of Cajal bodies (CBs) (indicated by *arrowheads *in Figure 4B, middle row). The CBs are functionally linked to the nucleolus and play a major role in the maturation of RNP, acting on the mRNA as well as the rRNA pathway [44].

These data demonstrate that in contrast to its yeast counterpart, hLsg1 localizes to the ER and to Cajal bodies in the nucleus.

### hLsg1 shuttles between the nucleus and the cytosol

**Figure 5:**
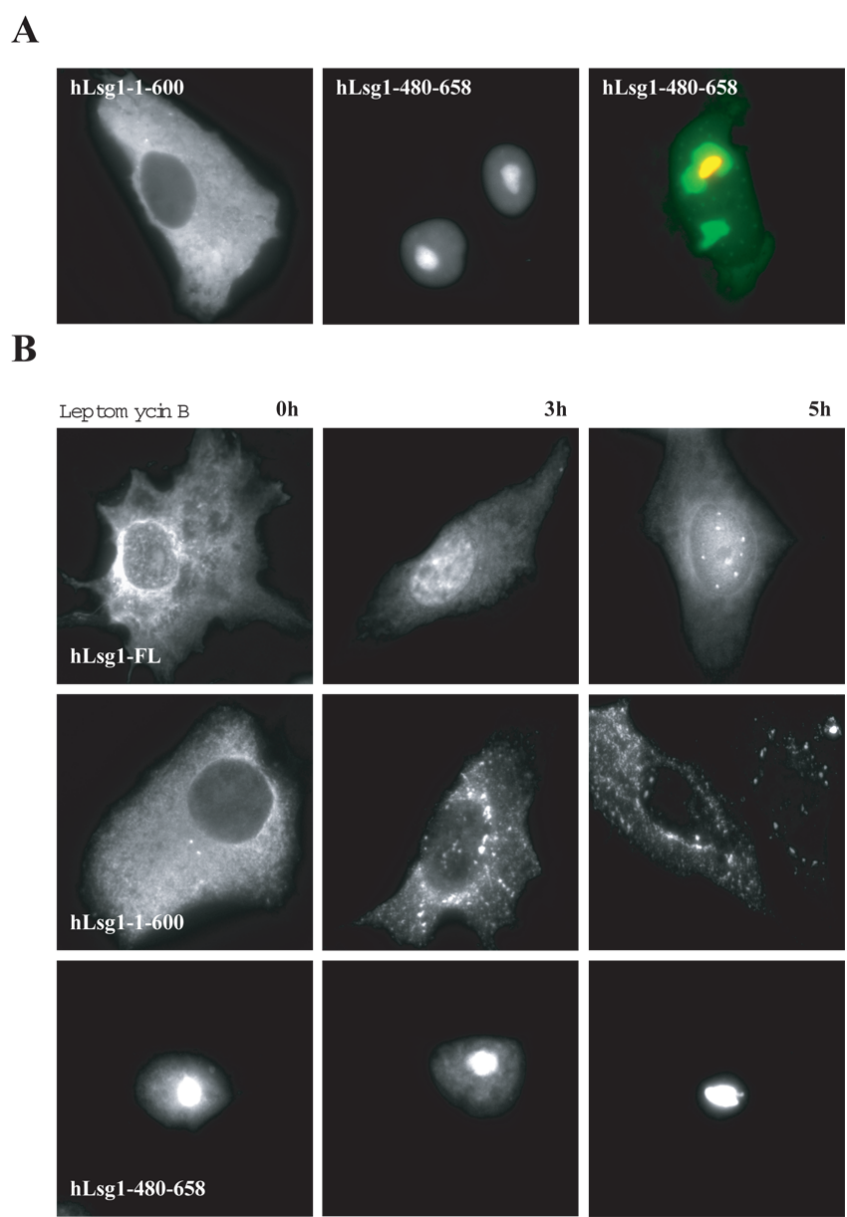
**hLsg1 localized to the nucleus upon Leptomycin B treatment. (A) Subcellular localization of YFP tagged hLsg1 mutants **– HeLa cells were transiently transfected with YFP-hLsg1-1-600 (1) or YFP-hLsg1-480-658 (2), or co-transfected with YFP-hLsg1-480-658 and SRP19-MRFP (3), and visualized by fluorescence microscopy. (B) **Localization of hLsg1 and mutants upon Leptomycin B treatment**. HeLa cells were transiently transfected with hLsg1-YFP (a), YFP-hLsg1-1-600 (b), or YFP-hLsg1-480-658 (c), treated with 15 nM Leptomycin B, fixed at 0 h, 3 h and 5 h, and visualized by fluorescence microscopy.

The dual localization of hLsg1 in the cytosol and nucleus suggests nucleocytoplasmic trafficking of the protein, possibly in relation to rRNA maturation. We constructed hLsg1 deletion mutants containing or excluding the putative the C-terminal NLS (YFP-hLsg1-1-600 and YFP-hLsg1-480-658) and transfected them into Hela cells (Figure 5A). While YFP-hLsg1-480-658 clearly localized in the nucleus, YFP-hLsg1-1-600, which contains no NLS, was excluded from the nucleus. Moreover, YFP-hLsg1-480-658 colocalized in the nucleus with SRP19-MRFP, a nucleolar marker [46], indicating that it sublocalizes to the nucleolus.

To determine whether hLsg1 shuttled between nucleus and cytosol via a CRM1-dependent nuclear export pathway, we transfected Vero cells with either hLsg1-YFP or hLsg1 deletion mutants and compared the localization of the fusion proteins after treatment with the CRM-1 nuclear export inhibitor Leptomycin B (LMB) (Figure 5B). Full-length hLsg1 (YFP-hLsg1) is LMB-sensitive (Figure 5B); so is its C-terminal counterpart hLsg1-CFP (data not shown). To confirm this, we performed the same experiment using the deletion mutants YFP-hLsg1-1-600 and YFP-hLsg1-480-658 as well as the full length YFP-hLsg1. We also took intermediate time points (3 h and 5 h) to obtain insights into the kinetics of hLsg1 shuttling. Interestingly, YFP-hLsg1 accumulates in the nucleus over an 8 h period, and at 5 h most of the transfected cells showed punctate labeling in the nucleus reminiscent of Cajal bodies. YFP-hLsg1-480-658 showed a permanent nuclear location and YFP-hLsg1-1-600 was constantly in the cytosol.

These data suggest that hLsg1 shuttles between the cytosol and Cajal bodies via a CRM1-dependent export mechanism.

## Discussion

Using database sequence similarity searches coupled with phylogenetic analysis, we were able to unite the circularly permuted GTPases into a family that we have named YRG for **Y**lqF **R**elated **G**TPases [see Additional files 1 and 2]. The YlqF protein family represents the largest subfamily of YRG expansion in eukarya, which is potentially involved in ribosome biogenesis.

Phylogenetic analysis defines ten GTPase subfamilies with a global phyletic distribution compatible with their presence in the last universal common ancestor (LUCA) of extant life forms [22]. An emerging concept suggests that these universal GTPases are necessary either for ribosome function or for transmitting information from the ribosome to downstream targets to generate specific cellular responses. These are associated with translation and include four translation factors, two OBG-like GTPases, the two signal-recognition-associated GTPases, the MRP subfamily of MinD-like ATPases and the YRG family. Here we have defined the YRG family for the first time as a eukaryotic expansion of the original Yawg/YlqF family [22] tightly coupled to the evolution of compartmentalization.

The YRG family was originally defined as a particular class of GTPases showing a circularly permuted structure, with the four GTPase motifs reorganized as G4 followed by G1, G2 and G3 (Figure 1A). This circular permutation is unique in the GTPase superfamily. However, we have shown that this inverted structure does not seem to affect GTPase activity or folding, in agreement with other studies [31,39]. Moreover, regarding the potential function of this family, it has been pointed out that most YRG members bind to the ribosome [YjeQ, [31]], are involved in the maturation of ribosomes or mitoribosomes [24,28,29,2], localize to compartments related to rRNA maturation [NGP, [1,39]], and are essential proteins (see Figure 3C and Additional file 2). Altogether, this indicates that YRG members have an essential role in ribosomal assembly.

**Figure 6:**
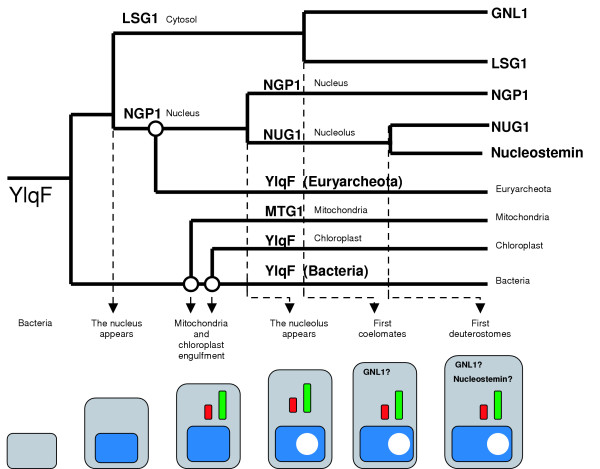
**The YRG family expands in relation to compartmentalization. **Scenario of the evolution of compartmentalization of the YRG members based on the phylogenetic tree in Figure 1.

Strikingly, we could find a member of the YRG family for every cellular compartment linked to ribosomes, including the chloroplast (Figure 1C), correlating with the expansion of the eukaryotic cell (Figure 1C). According to the phylogenetic tree of the family, the cytosolic form block (LSG1) is distinct from the nuclear form blocks (NOG2, NGP1, YawG), which later expanded into a nucleolar form (NUG1), in parallel with the incorporation of members upon engulfment of the future mitochondria (MTG1) that cluster within the YlqF branch as well as the future chloroplast (ChYlqF). Other events within the YlqF family included the appearance of a second cytosolic form upon speciation of the coelomates (GNL1), which may have had an equivalent in the plant lineage, since we observed a form of Lsg1 in *A. thaliana *(Figure 1B). Moreover, we observed the appearance of a second nucleolar form (Nucleostemin) upon speciation of the deuterostomes. Since Nucleostemin is involved in cell-cycle regulation in stem cells, we can hypothesize a direct mechanism of rRNA maturation in those highly specialized animal cells. We propose the following scenario for the evolution of the YRG family. First, a cytosolic founding member was duplicated upon the formation of a proto-nucleus, allowing the rRNA maturation pathway to be maintained (Figure 6). The second step included the engulfment of mitochondria and chloroplasts containing specific YRG forms involved in rRNA maturation in these compartments. The final step(s) involved the evolution of the cytosolic and nuclear members upon the specialization of the eukaryotic cell (nucleolus etc). This scenario accords with the work of Mans et al. [47], which showed by comparative genomics that a large set of proteins was involved in the formation and structure of the nuclear envelope and the pore complex: the nucleus evolved from a primordial prekaryote compartment and a primitive nuclear pore complex dependent on Ran and on Nug1p/Nug2p, a nucleolar YRG member.

Interestingly, hLsg1 is the only member of this family that shows a dual localization (cytosol/endoplasmic reticulum and Cajal Bodies). The cytosol contains huge numbers of ribosomes freely diffusing or bound to the endoplasmic reticulum, and is the main transit pathway for rRNA *en route *to the mitochondria or the chloroplast. Cajal Bodies are spherical nuclear bodies containing a variety of components including nucleolar proteins, snRNPs and SMN. They are dynamic structures functionally linked to the nucleolus, presumably involved in RNP maturation and related to gene expression [43,44]. Consistent with these data, one could hypothesize that hLsg1 is a regulator of the rRNA pathway that can relocate to Cajal Bodies and interact with specific factors such as nucleolar proteins. The observation that Leptomycin B treatment leads to accumulation of hLsg1 in the nucleus clearly indicates shuttling via a CRM1-dependent export pathway. We hypothesized that hLsg1 relocalizes from the cytosol to the nucleus in response to internal (e.g. cell cycle) or external (e.g. growth factor) stimuli. In this way, hLsg1 would act on the control of rRNA biosynthesis at its source: the nucleolus. In the future, these hypotheses will be tested for hLsg1 and for the other YRG family members to elucidate their role in rRNA biosynthesis and maturation.

## Conclusion

Using comparative genomics, we defined the YRG family as a unique group of circularly permuted GTPases. We suggest a potential function for this family, as well as a potential pathway by which the family members may act sequentially, following an evolutionary process linked to compartimentalization (Figure 6). A future goal will be to test this hypothesis experimentally and to dissect the molecular mechanisms of action of each member of the pathway.

## Methods

### Analysis of Lsg1p protein family

#### Database similarity searches

The translated sequence of the *Homo sapiens *gene FLJ11301 (GenBank accession no. NP_060855) was used to search the non-redundant protein database at the National Center for Biotechnology Information using the PSI-BLASTP program (15). Homologues were identified in *Homo sapiens *(GenBank accession identifier BAA92116), *Mus musculus *(XP_148574), *Danio rerio* (AAH66695), *Caenorhabditis elegans *(NP_490904), *Caenorhabditis briggsae *(CAE74467), *Drosophila melanogaster *(NP_569915), *Anopheles gambiae *(EAA13064), *Saccharomyces cerevisiae *(NP_011416), *Schizosaccaromyces pombe *(NP_593948), *Arabidopsis thaliana *(NP_172317), *Zea mays *(AAD41267), *Encephalitozoon cuniculi *(CAD26329), *Eremothecium gossypii *(NP_985506) and *Plasmodium falciparum *(NP_702181). The sequence corresponding to *Rattus norvegicus *had to be reconstructed using an insertion from *Mus musculus*, probably owing to an incorrect gene prediction (XP_213604).

#### Phylogenetic analysis

The 14 orthologous sequences were aligned using the ClustalW program [16]. PSI-BLAST searches on the NCBI protein database were performed using different representatives of the YRG family as seed, according to the bibliography, and were iterated until members of the closest subfamily were found in the list of hits. The sets of orthologous sequences were manually checked for sequence integrity and to clarify subfamily definitions. Progressively larger multiple sequence alignments were built by constructing multiple sequence alignments of each subfamily, which were manually polished and added together stepwise. At each step, the parts outside the central GTPase domain, which often showed no homology across subfamilies (and therefore should not be aligned), were trimmed to facilitate the production of the next multiple sequence alignment. The final multiple sequence alignment was used to produce the corresponding phylogenetic tree (excluding the non-aligned regions) using ClustalW. The full list of sequences used for the tree and their database identifiers are given as supplementary material [see Additional file 1].

### Cell culture, transfections, immunostaining and fluorescence microscopy

HeLa (ATCC CCL-2) and Vero (ATCC CCL-81) cells were cultured in Dulbecco's modified Eagle's medium (DMEM) supplemented with 10% FCS and penicillin/streptomycin at 37°C in an atmosphere of 5% CO_2_. Cells were seeded on to glass coverslips, Nunc plates or LabTek dishes and were transfected using Fugene6 (Roche) according to the manufacturer's protocols. For immunocytochemistry, transiently transfected HeLa cells were grown on coverslips and fixed in ice-cold methanol for 5 min at -20°C. The cells were then washed again and incubated in PBS for 20 min. Primary and secondary antibodies were diluted in PBS. The cells were incubated with primary antibodies followed by secondary antibodies for intervals of 30 min with three washing steps in between. The coverslips were then mounted in Mowiol on glass slides. Images of the stained cells were acquired using either a Zeiss Cell Observer System or a Leica AOBS confocal laser-scanning microscope.

### GTP binding and GTPase activity measurements

Nucleotide binding was measured by the filtration method. Recombinant proteins were incubated in 20 mM Tris-HCl pH 7.5, 1 mM DTT, 5 mM MgCl_2_, 10 mM EDTA, 0.5 g/l bovine serum albumin, (^3^H)GTP or (^3^H)GDP (7,7 Ci/mmol, Amersham-Pharmacia-Biotech) and cold 30 μM GTP or GDP. After incubation at 30°C for the indicated times, samples were diluted in 500 μl of ice-cold washing buffer (20 mM Tris-HCl pH 7.5, 25 mM MgCl_2 _and 100 mM NaCl) and applied to a nitrocellulose filter (0.45 μm, Millipore). The filters were rinsed with 4 × 4ml ice-cold washing buffer and the radioactivity retained on the filters was determined by scintillation counting.

GTPase activity measurement by HPLC was described by Ahmadian et al. 1999 [17].

### siRNAs transfection and western blotting

siRNA sequences were BLAST searched against the human genome to ensure that they were specific for hLsg1. The hLsg1 siRNA sequence showed no exact or near exact matches to any other sequence in the human genome and are therefore hLsg1-specific. siRNAs were synthesized by EUROGENTEC. hLsg1 siRNA (5'-UGGAGAGAAACUGCAAGACTT-3') targets nucleotides 506–524 of human hLsg1 relative to the first nucleotide of the start codon.

Cells were seeded into 12-well plates. Twenty-four hours later, they were transfected with 1.68 μg of siRNA per well (unless otherwise noted). Transfections were as described [28] with the following modifications. Additional OptiMEM (Invitrogen) was not added, and medium was removed before transfection and replaced with 400 μl of OptiMEM. Full-serum medium (unless otherwise noted) was added 4 h post-transfection. At the indicated times post-transfection, the cells were washed twice with PBS and detached from the plate with PBS EDTA. Whole cell extract was obtained by lysing the cells with RIPA buffer containing protease inhibitors and DTT. Protein concentrations were measured using the Bradford assay. Extracts were run on 8% polyacrylamide gels (12% for actin) and transferred to nitrocellulose membranes. The membranes were blocked overnight at 4°C in 1% non-fat dry milk (1 h at room temperature in 5% non-fat dry milk for actin), then probed with either rabbit polyclonal anti-hLsg1 or anti-actin (Santa Cruz Biotechnology) antibody for 1 h at room temperature (overnight at 4°C for actin), washed, and probed with a horseradish peroxidase-conjugated secondary antibody for 1 h at room temperature. Signals were detected using the ECL-Plus reagent (Amersham Biosciences).

## Authors' contributions

Reynaud E.G. carried out the molecular biology experiments, in collaboration with Ly T.B.N, the localization studies, the immunofluorescence and the cell biology studies. Andrade M.A. carried the complete phylogenetic studies of the YRG family and drafted part of the manuscript. Bonneau F. and Scheffzek K participated in the characterization of the Lsg1 GTPase activity. Knop M. participated in the design of, and performed part of, the yeast experiments. Pepperkok R. conceived the study and participated in its design and coordination. All authors read and approved the final manuscript.

## Supplementary Material

Additional File 1 **Complete Phylogenetic Tree of the YRG family**. A multiple alignment of sequences of the YqeH, YjeQ, EngA, and YlqF families. The alignment was produced using ClustalW followed by manual editing [16]. The tree was generated from the alignment using MrBayes v3 [45] (The root of the tree is the one given by the MrBayes output. Click here for file

Additional File 2 **The YRG family members**. The table compiles all the data collected from several datasets available for all the YRG members known so far. Click here for file
